# Diversity, in-vitro virulence traits and antifungal susceptibility pattern of gastrointestinal yeast flora of healthy poultry, *Gallus gallus domesticus*

**DOI:** 10.1186/s12866-017-1024-4

**Published:** 2017-05-15

**Authors:** Supram Hosuru Subramanya, Nawal Kishor Sharan, Bharat Prasad Baral, Deependra Hamal, Niranjan Nayak, Peralam Yegneswaran Prakash, Brijesh Sathian, Indira Bairy, Shishir Gokhale

**Affiliations:** 1 Manipal College of Medical Sciences, Pokhara, Nepal; 2 Kasturba Medical College, Manipal University, Udupi, India; 3 Melaka-Manipal Medical College, Manipal University, Udupi, India

**Keywords:** Poultry guano, Yeast, Antifungal resistance, Virulence factor, Gastrointestinal colonisation

## Abstract

**Background:**

Poultry farming and consumption of poultry (*Gallus gallus domesticus*) meat and eggs are common gastronomical practices worldwide. Till now, a detailed understanding about the gut colonisation of *Gallus gallus domesticus* by yeasts and their virulence properties and drug resistance patterns in available literature remain sparse. This study was undertaken to explore this prevalent issue.

**Results:**

A total of 103 specimens of fresh droppings of broiler chickens (commercial *G domesticus*) and domesticated chickens (domesticated *G domesticus*) were collected from the breeding sites. The isolates comprised of 29 (33%) *Debaryozyma hansenii* (*Candida famata)*, 12 (13.6%) *Sporothrix catenata* (*C. ciferrii*), 10 (11.4%) *C. albicans*, 8 (9.1%) *Diutnia catenulata* (*C. catenulate)*, 6 (6.8%) *C. tropicalis*, 3 (3.4%) *Candida acidothermophilum (C. krusei)*, 2 (2.3%) *C. pintolopesii*, 1 (1.1%) *C. parapsilosis*, 9 (10.2%) *Trichosporon* spp. *(T. moniliiforme, T. asahii)*, 4 (4.5%) *Geotrichum candidum*, 3 (3.4%) *Cryptococcus macerans* and 1 (1%) *Cystobasidium minuta (Rhodotorula minuta*). Virulence factors, measured among different yeast species, showed wide variability. Biofilm cells exhibited higher Minimum Inhibitory Concentration (MIC) values (μg/ml) than planktonic cells against all antifungal compounds tested: (fluconazole, 8–512 vs 0.031–16; amphotericin B, 0.5–64 vs 0.031–16; voriconazole 0.062–16 vs 0.062–8; caspofungin, 0.062–4 vs 0.031–1).

**Conclusions:**

The present work extends the current understanding of in vitro virulence factors and antifungal susceptibility pattern of gastrointestinal yeast flora of *G domesticus.* More studies with advanced techniques are needed to quantify the risk of spread of these potential pathogens to environment and human.

## Background

The prevalence and composition of yeast microbiota of the digestive tract vary considerably in animals and humans [1]. Gut microflora influences the health and well-being of host animals. Gut microflora can cause potentially life-threatening infections when the host’s biological homeostasis and immune resistance mechanisms are disrupted. The occurrence of different species of yeasts as natural gut residents of poultry has been documented [2, 3] but their ecological behaviour and yeast biome are poorly understood even though these birds are known to harbour pathogens with zoonotic potential [4].

There is growing concern that food derivatives from poultry sources may be an underestimated source of microorganisms with pathogenic potential. Certain species of yeasts commensal to a host species tend to have the pathogenic spectrum to others, including humans. Thus, natural yeast microbiota of animals may behave as pathogens in a suitable and conducive host. While moving between different niche and habitations, these can become vectors of virulence determinants and attain antimicrobial resistance [4, 5]. Several intrinsic factors contribute to the conversion of these organisms from harmless commensals to pathogens; the status of the host’s immune system, as well as putative virulence factors of the yeasts, play a major role in triggering infections and invading the host tissues [6]. Recently, Yuan Wu and colleagues [7] reported that fresh droppings from pigeons harboured several yeasts of medical importance and confirmed that pigeons could serve as potential reservoirs, carriers and spreaders of *Cryptococcus* and other medically important yeasts to humans. Psittacine birds as the source for dissemination of *Trichosporon, Candida* and several other yeasts to the environment and man was documented by Brilhante RS, et al. [8] who showed that these birds harboured potentially pathogenic yeasts throughout their gastrointestinal tract and in stool. Environmental pollution due to *Candida, Cryptococcus, Geotrichum, Rhodotorula* and *Trichosporon* from avian sources were in the recent past reported by Wojcik and co-workers [9], who were of the view that the presence of such organisms in the environment may pose a health risk to humans. Over and above, detection of multidrug resistant yeasts colonising the gastrointestinal tract (GIT) of synanthropic birds was of concern because these birds might be the reservoirs for transmission of drug resistant yeast infections to humans and such infections could be much severe in nature having greater potential to disseminate [7, 8]. In the above context, previous researchers reported the direct transmission of dermatomycoses to poultry workers and poultry handlers from various body parts and excreta of poultry [10–13]. Besides, another dimorphic fungus *Histoplasma capsulatum*, that can cause severe fatal disease in immunocompromised persons has been found in the droppings and body parts of certain avian species including chickens [14, 15] However, the role of yeast microbiota in poultry and its propensity for pathogenicity and infection is not yet well established. Undoubtedly, the close relationship between man and poultry may expose the human host to the poultry gut flora carrying virulence genes. Despite the well-known fact that domestic and wild birds may act as carriers of human pathogenic fungi, till today, clear evidence predicting poultry birds as reservoirs of drug-resistant and virulent yeasts, is lacking. It is, therefore, necessary to characterize the gastrointestinal (GI) yeast flora of poultry birds for a better understanding of the role of poultry in the possible evolution of emerging yeast infections. Considering the ever-expanding reports of opportunistic fungal infections and increasing popularity of poultry farming in Nepal, this study was conducted to characterize and evaluate in vitro virulence factors and antifungal susceptibility pattern among the GI yeast flora of both household and commercial poultry, *Gallus gallus domesticus.*

## Methods

### Sample collection

**Fig. 1:**
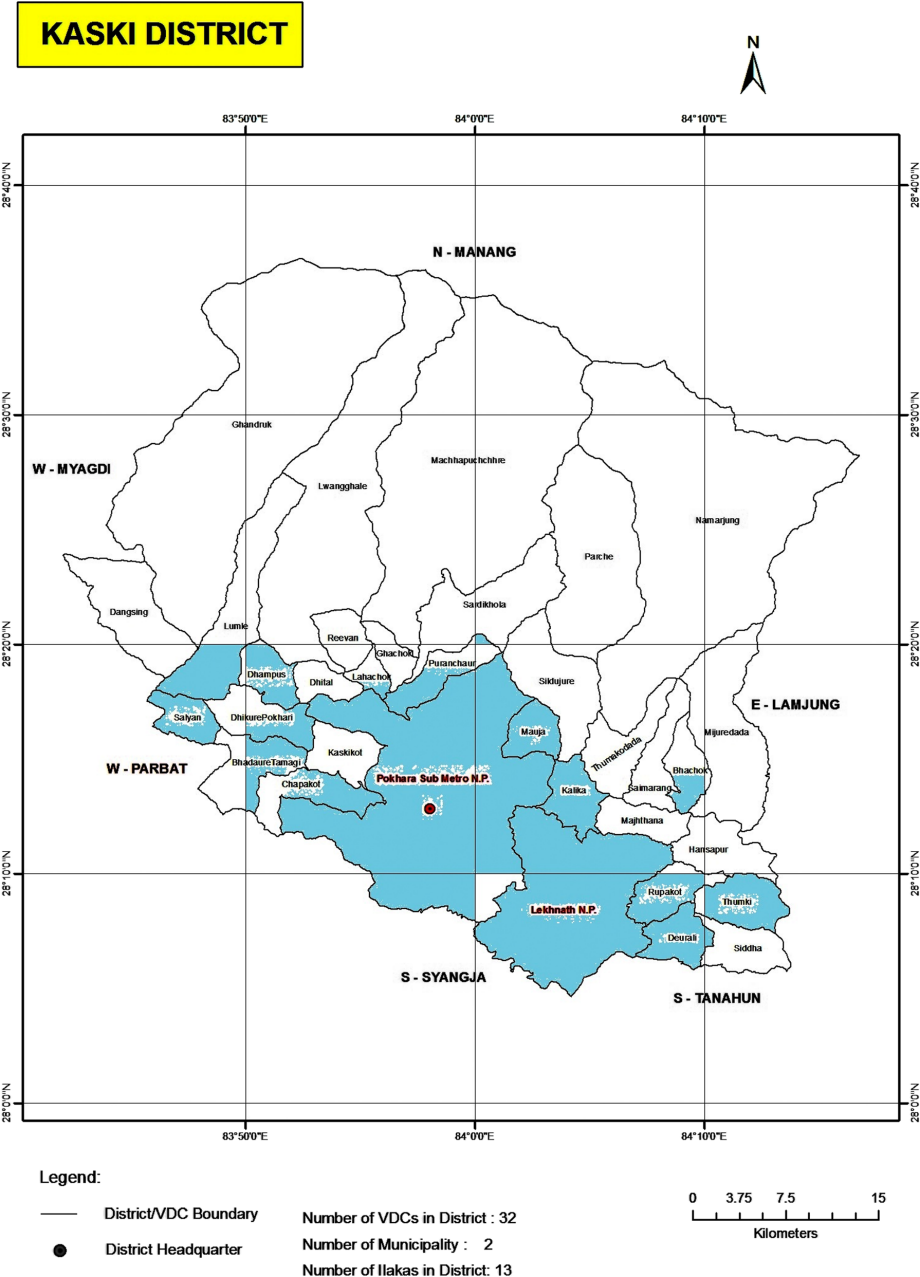
Locations of Kaski district from where samples were collected (highlighted in *blue*) http://lgcdp.gov.np/node/363

A total of 103 specimens of fresh bird droppings of adult broiler chickens (commercial *G domesticus*) and domesticated chickens (domesticated *G domesticus*) were collected from designated 45 sampling sites from commercial and local poultry breeders of Kaski district of western Nepal (Fig. 1). This region has scattered rural population of low socioeconomic background and unhygienic conditions. We selected those poultry farms that had adequate flock size, adequate husbandry practice and veterinary intervention, after obtaining factual information from the veterinarians that the reared flocks were healthy. Freshly passed poultry droppings from these sites were randomly collected with aseptic precautions, to avoid environmental contamination. The collected specimens were transported with ice packs to the microbiology laboratory of Manipal Teaching Hospital. The samples were processed within two hours of receipt in the laboratory.

## Culture and identification

Specimens were processed as per the recommended procedures [16]. Briefly, one gram of fecal sample was loaded into tubes containing 9 ml sterile normal saline and vortexed for a minute. Subsequently, a serial tenfold dilution starting from 10^−1^ to 10^−4^ were carried out, and 100 μl aliquots were plated onto Sabouraud Dextrose Agar (SDA), supplemented with chloramphenicol (0.05 g/l; HiMedia, India). Total viable counts were determined after three days of incubation at 37^0^ C. Colony count was expressed as CFU per gram of fecal sample. The yeast isolates were identified by conventional techniques including morphological, physiological, biochemical and vitek based identification methods [16, 17].

*Trichosporon* species not identifiable by conventional methods were confirmed by amplification and sequencing of the intergenic spacer 1 (IGS1) regions of rDNA [18, 19] at National Culture Collection of Pathogenic Fungi (NCCPF), Postgraduate Institute of Medical Education and Research, Chandigarh, India**.**

### Extracellular enzymatic and hemolytic activity assays

All assays were performed as described earlier [20–25]. For the analysis of phospholipase, aspartyl proteinase, hemolysin and esterase, yeast cells were suspended in sterile normal saline at a concentration of 1 × 10^5^ CFU/ml, and 5 μl of yeast suspension were charged on the 6 mm filter paper disc and inoculated on appropriate medium. Phospholipase activity was assayed on egg yolk medium (65 g/l SDA, 3% *w*/*v* glucose, 1 M NaCl, 5 mM CaCl_2_ and 8% egg yolk emulsion) [22]. Aspartyl proteinase activity was carried out on bovine serum albumin (BSA) agar (1.17% *w*/*v* yeast carbon base, 0.01% *w*/*v* yeast extract, 0.2% *w*/*v* BSA and 1.12% agar) as described previously [23]. Phospholipase and proteinase activities were determined to be positive if there was formation of precipitation halo around the fungal growth. Hemolytic activity assays were performed on SDA, incorporated with sheep blood (3% *w*/*v* glucose and 10% *v*/v sheep blood) [21] and were considered positive by the presence of a translucent halo around the inoculation site, viewed under transmitted light. For the assessment of DNAse activity, strains were directly spot-inoculated on DNAse test agar (HiMedia, India) and interpreted according to the instructions of the manufacturer. *Staphylococcus aureus* ATCC 25923 was used as a positive control in the DNAase test. Esterase activity was evaluated on Tween 80 medium (1% *w*/*v* Bacto peptone, 0.5%*w*/*v* NaCl, 0.01% *w*/*v* CaCl2, 1.5% *w*/*v* agar and 0.5% *v*/v Tween 80) [24]. Positivity for esterase was indicated by the presence of opaque crystals around the colony, visible against transmitted light. For all assays, the plates were incubated at 37^0^ C for 5–7 days. Enzymatic activities (phospholipase, aspartyl proteinase, and esterase) were expressed as Pz values, which measured the diametrical ratio of the colony to halo. Activity was thus categorized as “very strong” (Pz ≤ 0.69), “strong” (Pz =0.70–0.79), “mild” (Pz = 0.80–0.89), “weak” (Pz = 0.90–0.99), or “negative” (Pz = 1.0) [25]. To test the oxidative stress tolerance, the yeast cell suspensions were treated for 1 h at 37 °C with hydrogen peroxide at the concentrations of 5, 10, 20, 40 and 50 mM. A 5 μL of the suspension was spot inoculated on SDA plates. Macro-morphology of growth (diameter and number of micro colonies) was monitored after 48-h of incubation at 37 °C and was compared to the yeast growth on control plates with no H_2_O_2_ exposure [26]. Cell surface hydrophobicity (CSH) was demonstrated as follows: 48-h old yeast culture on yeast extract peptone dextrose agar (YEPD) was adjusted to an optical density of 1.0 at 520 nm. One millilitre of xylene was added to each suspension; the test tubes were placed in a water bath at 37 °C for ten minutes to equilibrate, then vortexed for 30 s, and finally returned to the water bath for 30 min to allow the xylene and aqueous phases to separate. The absorbance of the aqueous phase was measured at 520 nm. Cell surface hydrophobicity was expressed as the percentage reduction in optical density of the test suspension compared with control. The greater the change in absorbance, the more was the hydrophobicity [20]. Activity was thus categorized as “very strong” (>30%), “strong” (20–29.9%), “mild” (10–19.9%), “weak” (0.1–9.99%), or “negative” (< 0.1%). All assays were performed in triplicate on separate occasions.

### Biofilm assay

The yeast isolates grown overnight on YEPD broth at 37^0^ C were harvested, washed twice with sterile PBS and then re-suspended in YEPD broth, to a concentration of 10^5^ CFU/ml. Biofilms were formed by pipetting 200ul of the standardized cell suspension into commercially available pre-sterilized polystyrene 96 wells tissue culture plates (HiMedia, India) and incubating at 37^0^ C for 90 min (adhesion phase). Wells were, then, washed twice with sterile phosphate-buffered saline (PBS) to remove non-adherent cells and then refilled with 200 μl of sterile YEPD broth, re-incubated for further 48 h at 37 °C, replacing the medium at 24 h of incubation (biofilm formation phase). At the end of the whole process, the medium was aspirated and non-adherent cells were removed by washing the wells thrice in PBS. Known biofilm producer and non-biofilm producer *Candida* strains served as positive and negative controls respectively.

### Quantification of biofilm

Quantitative biofilm assessments were performed by crystal violet staining method (staining for biomass) [27] and colorimetric measurement based on sodium 39-[1-(phenylamino-carbonyl)-3,4-tetrazolium]-bis (4-methoxy-6-nitro) benzene sulfonic acid hydrate (XTT) reduction (metabolic activity) as previously described [28].

### Antifungal susceptibility testing against planktonic and biofilm-forming cells

Assay conditions of the Clinical and Laboratory Standards Institute (CLSI) broth micro dilution method [29] were adopted to evaluate the response of planktonic fungal cells to the following drugs: fluconazole (FLC), voriconazole (VRC), caspofungin (CFG) and amphotericin B deoxycholate (AMB). Antifungal compounds were obtained as pure powders from the manufacturer, Sigma-Aldrich Laborchemikalien GmbH, Germany. *Candida parapsilosis* ATCC 22019 and *Candida krusei* ATCC 6258 were used as controls. Minimum Inhibitory Concentrations (MIC) of the drugs on planktonic cells were determined by visual readings after 48 h of incubation based on the lowest concentration capable of inhibiting 50% of cell growth for azoles and 100% for AMB and CFG.

Susceptibility tests for biofilm-forming cells were performed following the protocol previously described by Melo et al. [30]. Biofilms were grown for 24 h before replacement of the medium with fresh YEPD supplemented with the antifungals at the following concentrations: FLC (2–512 μg/ml), CFG (0.5-64 μg/ml), VRC (0.5–64 μg/ml) and AMB (0.5–64 μg/ml). Following a further incubation for 48 h, biofilms were quantified using the XTT reduction assay. For the XTT reduction assay, a solution containing 200 ml PBS with 12 ml 5:1 [XTT (1 mg/ml): Menadione (0.4 mM)] was used. The plate was incubated for 2 h at 37 °C to allow XTT metabolization. Thereafter 100 μl of this solution was transferred to another microplate, and the absorbance was read spectrophotometrically at (492 nm/620 nm [read/reference]). Minimum Inhibitory Concentrations of biofilm cells was determined as the lowest concentration of antifungal agent causing a 100% reduction in metabolic activity.

### Statistical analysis

Descriptive and inferential statistics were used for analyzing the data entered in Microsoft Excel 2010 by Statistical Analysis System (SAS) and Origin Pro 2016. Variation of colony count (CFU/g) in different yeast species was obtained by using minimum, maximum, mean and box plot. Descriptive analysis was performed to determine the frequency of the virulence factors viz., biofilm, DNase, hemolysin, esterase, aspartyl proteinase, phospholipase, CSH, SOD among yeast isolates. Spearman correlation was used to correlate various virulence factors. In vitro activity of antifungal drugs against planktonic cells, and biofilm cells were estimated with geometrical mean, 90th percentile, 50th percentile, and range. The “p” value of <0.01 was considered statistically significant.

## Results

A total of 103 poultry dropping specimens were collected from 42 commercial and 61 local breeders of Kaski district of western Nepal. Twelve different yeast species were grown on 84 samples (81.5%), 43 yeast strains were isolated from broiler chickens and 45 yeast strains were isolated from domesticated chickens. As depicted in Table 1, the isolates comprised of 29 (33%) *Debaryozyma hansenii* (*Candida famata)*, 12 (13.6%) *Sporothrix catenata* (*C. ciferrii*), 10 (11.4%) *C. albicans*, 8 (9.1%) *Diutnia catenulata* (*C. catenulate)*, 6 (6.8%) *C. tropicalis*, 3 (3.4%) *Candida acidothermophilum (C. krusei)*, 2 (2.3%) *C. pintolopesii*, 1 (1.1%) *C. parapsilosis*, 9 (10.2%) *Trichosporon* spp. *(T. moniliiforme, T. asahii)*, 4 (4.5%) *Geotrichum candidum*, 3 (3.4%) *Cryptococcus macerans* and 1 (1%) *Cystobasidium minuta (Rhodotorula minuta*).

**Table 1:** Biofilm and other virulence determinants of yeast isolated from *Gallus gallus domesticus* droppings

Virulence determinants		Yeast isolates											
		*C. albicans (n = 7* ^*c*^ *+ 3* ^*d*^ *)*	*C. catenulate (n = 3* ^*c*^ *+ 5* ^*d*^ *)*	*C. tropicalis (n = 3* ^*c*^ *+ 3* ^*d*^ *)*	*C. famata (n = 12* ^*c*^ *+ 17* ^*d*^ *)*	*C. ciferrii (n = 8* ^*c*^ *+ 4* ^*d*^ *)*	*C. pintolopesii (n = 0* ^*c*^ *+ 2* ^*d*^ *)*	*C. parapsilosis (n = 0* ^*c*^ *+ 1* ^*d*^ *)*	*C. krusei (n = 3* ^*c*^ *+ 0* ^*d*^ *)*	*C.* macerans (*n* = 1^*c*^ *+ 2* ^*d*^)	*G. candidum (n = 0* ^*c*^ *+ 4* ^*d*^ *)*	*Trichosporon spp. (n = 5* ^*c*^ *+ 4* ^*d*^ *)*	*R. minuta (n = 1* ^*c*^ *+ 0* ^*d*^ *)*
Colony count (CFU/g)		Min = 2 × 10^6^ Max = 7 × 10^6^ M = 5 × 10^6^	Min = 2.7 × 10^6^ Max = 1.2 × 10^7^ M = 6.6 × 10^6^	Min = 3 × 10^6^ Max = 2.3 × 10^7^ M = 1.1 × 10^7^	Min = 1.1× ×10^6^ Max = 1.2 × 10^7^ M = 5 × 10^6^	Min = 3 × 10^6^ Max = 1.9 × 10^7^ M = 9 × 10^6^	Min = 3 × 10^6^ Max = 1.6 × 10^7^ M = 9.5 × 10^6^	Load = 1.1 × 10^7^	Min = 1.1 × 10^6^ Max = 5 × 10^6^ M = 3 × 10^6^	Min = 3 × 10^6^ Max = 1.6 × 10^7^ M = 8.3 × 10^6^	Min = 2 × 10^6^ Max = 4 × 10^6^ M = 3.3 × 10^6^	Min = 4 × 10^5^ Max = 5 × 10^6^ M = 3.3 × 10^6^	Load = 2 × 10^6^
Biofilm Positive		5	4	1	10	7	0	0	1	3	4	3	0
Biofilm Negative		5	4	5	19	5	2	1	2	0	0	6	1
DNAse Positive		1	3	0	4	1	0	0	0	1	0	1	0
DNAse Negative		9	5	6	25	11	2	1	3	2	4	8	1
Hemolysin Positive		0	0	0	0	0	0	0	0	0	0	0	0
Hemolysin Negative		10	8	6	29	12	2	1	3	3	4	9	1
Esterase	Pz value^a^												
Very strong	≤0.69	6	1	0	5	2	0	1	2	1	1	0	0
Strong	0.70–0.79	0	0	0	0	1	0	0	0	0	0	0	0
Mild	10–19.9	0	0	0	0	1	0	0	0	0	0	1	1
Weak	0.90–0.99	0	0	0	0	0	0	0	0	0	0	0	0
Negative	>/= 1.0	4	7	6	24	8	2	0	1	2	3	8	0
Aspartyl proteinase	Pz value^a^												
Very strong	≤0.69	2	0	1	8	0	1	1	1	0	0	0	0
Strong	0.70–0.79	2	1	1	3	0	0	0	1	1	0	1	1
Mild	0.80–0.90	0	0	0	0	0	0	0	0	0	0	0	0
Weak	0.90–0.99	0	0	0	0	0	0	0	0	0	0	0	0
Negative	>/= 1.0	6	7	4	18	12	1	0	1	2	4	8	0
Phospholipase	Pz value^a^												
Very strong	≤0.69	0	0	0	0	0	0	0	0	1	0	0	0
Strong	0.70–0.79	1	1	0	0	0	0	0	0	0	0	0	0
Mild	10–19.9	1	0	0	0	0	0	0	0	0	0	0	0
Weak	0.90–0.99	0	0	0	0	0	0	0	0	0	0	0	0
Negative	>/= 1.0	8	7	6	29	2	2	1	3	2	4	9	1
CSH	%												
Very strong	>30	6	5	4	12	10	1	0	2	2	2	6	0
Strong	20–29.9	2	0	0	4	0	1	1	1	0	0	1	0
Mild	10–19.9	1	2	0	5	1	0	0	0	1	0	0	1
Weak	0.1–9.99	1	1	2	8	1	0	0	0	0	2	2	0
Negative	< 0.1	0	0	0	0	0	0	0	0	0	0	0	0
SOD	H_2_O_2_ mMol												
Very strong	50	4	3	1	11	5	0	1	2	2	4	5	0
Strong	40	1	1	1	1	2	0	0	1	0	0	1	0
Strong	30	1	0	0	3	0	0	0	0	0	0	0	0
Mild	20	0	2	0	1	1	0	0	0	1	0	1	0
Mild	10	2	2	2	4	3	1	0	0	0	0	0	0
Weak	5	2	0	1	6	0	1	0	0	0	0	2	0
Negative	No growth	0	0	1	3	1	0	0	0	0	0	0	1

^a^Pz value: the ratio of the colony diameter to the diameter of the activity halo; Min-minimum; Max-maximum; M-mean value; ^c^commercial *G domesticus*; ^d^domesticated *G domesticus*

### Fungal burden (CFU/g) in the poultry GIT

**Fig. 2:**
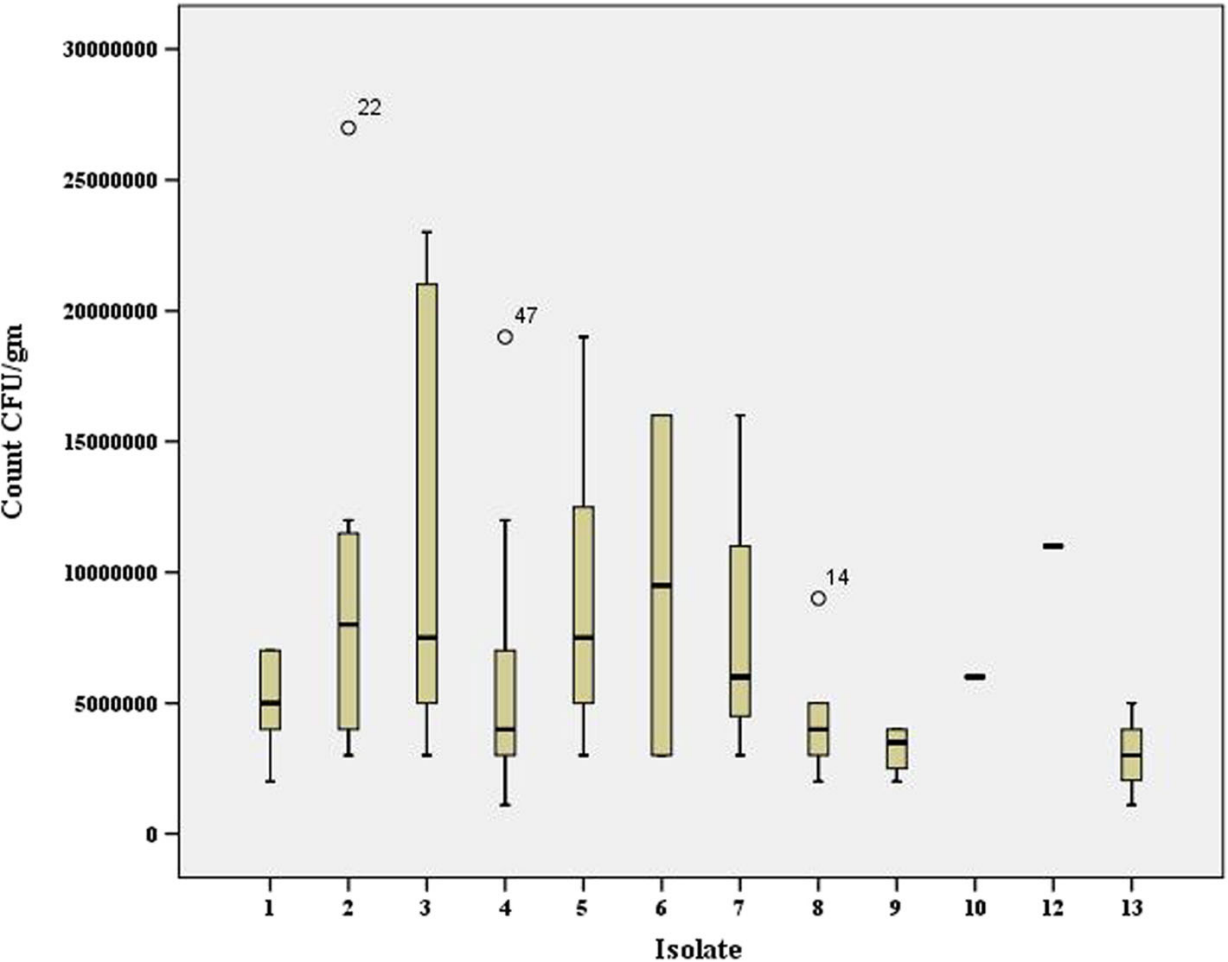
A box plot representation of the CFU/g median values of different yeasts isolated from *Gallus gallus domesticus.* Legend: 1: *C. albicans*, 2: *D. catenulate* (*C catenulate*)*,* 3: *C.* tropicalis, 4*: D. hansenii* (*C.* famata)*,* 5: *S. catenata* (*C* ciferrii)*,* 6: *C. pintolopesii,* 7: *Cryptococcus macerans,* 8: *Trichosporon* spp*.* 9: *G candidum* 10: *Cystobasidium minuta* (*Rhodotorula minuta*)*,* 12: *C parapsilosis*, 13: *C*. *acidothermophilum* (*C.* krusei)

Colony counts [mean, median and range interval (minimum- maximum) CFU/g] have been depicted in Fig. 2 and Table 1. The overall median CFU for the 88 yeast isolates was 0.5X10^7^ CFU/g (interquartile range (IQR) 0.5X10^7^). It was interesting to note that both mean colony count and colony count range for *C. albicans* (mean 5 × 10^6^ CFU/g, range 2 × 10^6^- 7 × 10^6^) were comparable to those for *D. hansenii* (mean 5 × 10^6^ CFU/g, range 1.1 × 10^6^–1.2 × 10^6^) and *C*. *acidothermophilum* (mean: 3 × 10^6^ CFU/g, range: 1.1 × 10^6^-5 × 10^6^); but were found to be much lower in comparison to the respective values for other yeasts and non albicans *Candida* species (*D. catenulata*, mean: 6.6 × 10^6^, range: 2.7 × 10^6^–1.2 × 10^7^; *C. tropicalis*, mean:1.1 × 10^7^, range:3 × 10^6^–2.3 × 10^7^; *S. catenata,* mean: 9 × 10^6^, range:3 × 10^6^–1.9 × 10^7^; *C. pintolopesii*, mean; 9.5 × 10^6^, range: 3 × 10^6^–1.6 × 10^7^, *Cryptococcus macerans,* mean: 8.3 × 10^6^, range: 3 × 10^6^–1.6 × 10^7^). The overall fungal burdens in the case of *C. pintolopesii*, *C. tropicalis*, and *C. ciferrii* were, however, higher compared with *C. albicans, C. parapsilosis, C. krusei* and other yeasts, such as *Geotrichum candidum*, *Trichosporon* species. Such statistical comparison could not be drawn in cases of *C. parapsilosis* and *C. minuta* as those were single isolates.

### Isolates were able to produce hydrolytic enzymes and other phenotypic virulence factors

Out of 88 yeast isolates, 38 isolates (43.1%) were biofilm producers in term of both biomass production and metabolic activity of sessile cells*. Rhodotorula minuta*, *C. pintolopesii* did not show in vitro biofilm activity. Most of the strains (56.8%; 50/88) displayed high CSH and superoxide dismutase (46.3%; 38/82) activity. Similarly, high to medium proteinase (28.4%) and esterase activity (26.1%) were observed among the isolates. DNase was detected in 11 isolates (12.5%). None of the isolates was positive for hemolysin. Similarly, the majority of yeast species (95.5%) did not produce any detectable phospholipase. The results for virulence determinants are summarized in Table 1.

### Observation of statistical relationships between the occurrences of different virulence factors

Overall, the relationship found among the different virulence factors of the yeast isolates was minimal (Table 2). However, an observable relationship was detected between the Pz values of phospholipase with Pz value of proteinase; CSH percentage with Pz values Esterase and Pz values phospholipase with Pz values DNAs (Spearman’s correlation coefficient of 0.407 (*p* = 0.000), 0.237 (*p* = 0.026), 0.240 (*p* = 0.024) respectively).

**Table 2:** Spearman’s correlation coefficient for in-vitro virulence factor measurements of yeast isolate

Assay Parameters		DNAse (Pz)	Biofilm (OD)	CSH (%)	SOD (mMol)	Proteniase (Pz)	Phospholipase (Pz)	Esterase (Pz)
DNAse (Pz)	Spearman’s Correlation	1	0.133	0.158	0.035	−0.054	0.240	0.188
	*P* value	--	0.216	0.141	0.749	0.615	0.024	0.079
Biofilm (OD)	Spearman’s Correlation	0.133	1	−0.008	−0.023	0.140	0.126	0.002
	*P* value	0.216	--	0.941	0.835	0.195	0.243	0.983
CSH (%)	Spearman’s Correlation	0.158	−0.008	1	−0.029	0.020	0.059	0.237^a^
	*P* value	0.141	0.941	--	0.787	0.850	0.586	0.026
SOD (mMol)	Spearman’s Correlation	0.035	−0.023	−0.029	1	−0.187	−0.092	0.101
	*P* value	0.749	0.835	0.787	--	0.080	0.396	0.351
Proteinase (Pz)	Spearman’s Correlation	−0.054	0.140	0.020	−0.187	1	0.407^b^	0.127
	*P* value	0.615	0.195	0.850	0.080	--	0.000	0.237
Phospholipase (Pz)	Spearman’s Correlation	0.240^a^	0.126	0.059	−0.092	0.407^b^	1	0.095
	*P* value	0.024	0.243	0.586	0.396	0.000	--	0.381
Esterase (Pz)	Spearman’s Correlation	0.188	0.002	0.237	0.101	0.127	0.095	1
	*P* value	0.079	0.983	0.026	0.351	0.237	0.381	--

^a^Correlation is significant at the 0.05 level; ^b^Correlation is significant at the 0.01 level

### Biofilm-forming cells had higher MIC values against antifungal agents

Table 3 summarizes the planktonic MIC50, MIC90, and MIC ranges (μg/ml) and the geometric mean MICs (GM) obtained for the 12-yeast species against fluconazole, amphotericin B, voriconazole, and caspofungin. Minimum Inhibitory Concentration values of fluconazole, amphotericin B were found to be higher for all fungal isolates tested. The respective values for the biofilm cells were noted to be comparatively higher than those detected in the planktonic cells (Table 3), suggesting thereby that biofilm in an in vivo situation could be less amenable to a broad range of antifungal agents in clinical use. Table 4 shows the MIC values for all antifungals tested against biofilm cells.

**Table 3:** In vitro activity of antifungal drugs against planktonic cells

Species (No. of isolates)	MIC (μg/ml) of Planktonic cells															
	Fluconazole				Amphotericin B				Voriconazole				Caspofungin			
	Interval	MIC 50	MIC 90	GM	Interval	MIC50	MIC90	GM	Interval	MIC50	MIC90	GM	Interval	MIC 50	MIC90	GM
*C. albicans* (10)	0.03–16	4.5	16	2	0.03–8	0.37	2.6	0.4	0.125–8	0.37	8	0.61	0.03–0.5	0.125	0.5	0.12
*D. catenulata* (8)	0.5–16	5	16	3.3	0.06–16	0.25	4.9	0.27	0.125–0.5	0.5	0.5	0.29	0.0625–0.5	0.125	0.325	0.14
*C. tropicalis* (6)	4–16	8	12	7.12	0.03–16	2.5	16	1	0.125–8	0.5	6	0.89	0.125–.25	0.125	0.25	0.15
*D. hansenii* (29)	0.03–16	1	9.6	0.952	0.03–16	0.25	1	0.28	0.06–1	0.25	0.5	0.20	0.03–1	0.125	0.5	0.17
*S. catenata* (12)	0.13–16	4	8	2.5	0.03–16	0.18	14.8	0.35	0.125–1	0.5	0.5	0.37	0.125–1	0.25	0.25	0.21
*C. pintolopesii* (2)	0.03–8	-	-	0.49	0.03–1	-	-	0.17	0.125–0.5	-	-	0.25	0.5–0.5	-	-	0.5
*C. parapsilosis* (1)	2	-	-	-	0.5	-	-	-	0.25	-	-	-	0.0625	-	-	-
*C. acidothermophilum* (3)	8–16	-	-	12.7	0.03–0.25	-	-	0.09	0.062–4	-	-	0.39	0.031–0.125	-	-	0.06
*Cryptococus macerans* (3)	0.5–1	-	-	0.79	0.13–0.5	-	-	0.31	0.5–4	-	-	1	0.0313–0.5	-	-	0.15
*Trichosporon spp*. (9)	0.03–16	4	9.6	2.92	0.25–16	1	9.6	1.36	0.120.5	0.25	0.5	0.27	0.062–0.5	0.125	0.3	0.14
*G. candidum* (4)	0.5–8	-	-	2	0.03–2	-	-	0.29	0.125–0.5	-	-	0.29	0.031–0.25	-	-	0.14
*Cystobasidium minuta* (1)	1	-	-	-	0.125	-	-	-	0.25	-	-	-	0.125	-	-	-

MIC50: Minimal inhibitory concentration capable of inhibiting the growth of 50% of isolates; MIC90: Minimal inhibitory concentration capable of inhibiting the growth of 90% of isolates; GM: Geometric Mean MIC

**Table 4:** In vitro activity of antifungal drugs against biofilm forming cells

Yeast species (No. of isolates)	MIC (mg/ml) of biofilm forming cells															
	Fluconazole				Amphotericin B				Voriconazole				Caspofungin			
	Interval	MIC50	MIC90	GM	Interval	MIC50	MIC90	GM	Interval	MIC50	MIC90	GM	Interval	MIC50	MIC90	GM
*C. albicans* (5)	8–256	128	204.8	64	0.5–4	1	3.2	1.3	0.5–8	4	8	2.29	0.062–0.5	0.125	0.5	0.18
*D. catenulata* (4)	16–256	-	-	45.2	0.5–8	-	-	2	1–2	-	-	1.18	0.125–0.5	-	-	0.25
*C. tropicalis* (1)	128	-	-	-	16	-	-	-	1	-	-	-	8	-	-	-
*D. hansenii* (10)	8–512	33	166.4	42.4	0.5–16	1	5.2	1.62	0.125–4	0.5	1.3	0.65	0.125–1	0.5	0.5	0.37
*S. catenata* (7)	8–512	64	281.6	47.5	1–16	2	8	2.2	0.5–16	0.5	7	1	0.5–4	0.5	2.2	0.74
*C. acidothermophilum* (1)	128	-	-	-	1	-	-	-	8	-	-	-	0.0625	-	-	-
*Trichosporon spp.* (3)	16–256	-	-	50.7	4–16	-	-	6.3	0.5–1	-	-	0.62	0.125–0.5	-	-	0.31
*G. candidum* (4)	16–128	-	-	53.8	1–2	-	-	1.18	0.5–1	-	-	0.59	0.125–1	-	-	0.42
*Cryptococus macerans* (3)	8–32	-	-	16	1–8	-	-	2.5	1–8	-	-	2	0.125–1	-	-	0.39

## Discussion

It is well known that opportunistic fungal infections were one of the emerging problems globally [31, 32]. Organisms that were once relegated as innocuous inhabitants of the environment have now emerged as potential opportunistic pathogens with the ability to colonize and infect susceptible hosts. In the 1980s, the aetiology of invasive yeast infection in humans was restricted only to those caused by *C albicans*. In the recent years, non-albicans *Candida* species accounted for >50% of invasive fungal infections [33]. *Candida* bloodstream infections were also recorded to be quite high due to non- albicans *Candida* species, accounting for more than 28% of cases [34]. Over and above, invasive infections due to other rare yeasts, such as *Trichosporon spp.*, *Geotrichum spp.*, *Cryptococcus* other than *C neoformans* and *Rhodotorula spp.* were recently reported [35–38]. A few studies suggested that GI colonization could be one of the potential sources for deep-seated yeast infections [39, 40].

Many animals including poultry were in the recent past, recognized as carriers of livestock-associated pathogens that could on a number of occasions cause disease in the human host [41, 42]. The present study demonstrated diverse generic groups of yeasts with high MIC values. Prevalence of yeast flora in the digestive tract of these birds revealed higher colonization by *Candida* other than *C albicans*. This is contrary to the observation of Shokri H et al. [42] and Lord et al. [3] who noticed the highest prevalence of *C albicans* among the gut flora of broiler chickens. The fungal burden as determined by our study was as high as 0.5 × 10^7^ CFU/g in terms of statistical mean values. Comparatively, the much lower fungal burden was documented in similar other studies [42]. Such variations in the gut colonisation by different *Candida* species as observed could be attributed to dietetic and environmental factors, hygienic conditions, and other ecological factors at different geographical locations [43]. The birds under particular combinations of diet or under stress could have higher level of corticosteroids that would affect the immune system resulting in higher gut colonisation [43–45]. It is difficult to predict which factors the present flock had been exposed to. High fungal burden in the gut microbiota of the poultry in our study suggests greater chance of dissemination of the gut flora to other poultries, human beings and their environments [8, 46]. This proposition is substantiated by other studies [7–9].

Colonisation rates of *Trichosporon* spp*., G candidum, Rhodotorula spp.* and *S cerevisiae* was shown to be 5.5%, 4.6%, 3.3% and 0.5% respectively, in a recent study conducted by Shokri et al. [42]. Our results documented higher colonisation rates of *Trichosporon spp., Geotrichum candidum,* and *Rhodotorula minuta* as 10.2%, 4.5%, and 1.1% respectively. This difference could be due to the more sensitive and specific molecular techniques and automated identification systems deployed in our study. It was interesting to find *Cryptococcus macerans* among the gut flora of three of the birds. *Cryptococcus neoformans* is known to colonise the GIT of many avian species, especially pigeons with potential threat to the human from the dried and desiccated droppings of these birds [7]. The zoonotic potential of non-neoformans *Cryptococcus* species from poultry gut flora is not well studied. Detecting these organisms in the gut flora raises the possibility of colonisation by *C. neoformans* as well. This plausibility calls for strict vigilance.

Most of the isolated organisms in the present study are documented human pathogens with evolving zoonotic potential. Virulence factors play a significant role in colonising the host evading the host defence there by contributing to the pathogenicity. These include the ability to adhere to surfaces and secretion of various hydrolytic enzymes [47, 48]. In the present study, 46.3% of the isolates produced SOD, and 56.8% possessed CSH. This is in agreement with the observations of earlier workers who proposed that CSH was a prerequisite for biofilm formation [49]. We observed that, 43.1% of the total yeasts and 39.4% *Candida* species were biofilm producers. Among all the biofilm producing *Candida* species, non-albicans *Candida* species alone accounted for 82.1% (23/28). As has been reported, biofilm formation on indwelling medical devices, especially by *Candida* species and *Trichosporon* species, is increasingly recognized for evading the host immunity and often leading to treatment failure [3, 50–54]. Detecting such high rate of biofilm producing non-albicans *Candida* is of utmost significance.

Variation in the production of hydrolytic enzymes among different species of yeasts was observed; most of them being able to exhibit in-vitro high-level CSH and SOD. High degree of positive concordance among different phenotypic enzymatic markers was not detected despite an observable relationship of phospholipase with proteinase, CSH with esterase and phospholipase with DNAs. Samaranayake et al. [20], noted the positive correlation between CSH and adhesion of *C. krusei* isolates onto HeLa cells and were of the view that this attribute along with other cell surface features might determine the hierarchy of virulence among different *Candida* species. Despite lack of significant correlation between different virulence markers as stated above, production of biofilm could be singled out as the sole pathogenic biomarker that was exhibited by 43.1% of all yeasts including 39.4% of *Candida* species. This supports the recent proposition of the role of biofilm in pathogenicity of *Candida* [50, 51]. All phenotypic characters may not necessarily be attributes of pathogenicity of *Candida* and other yeasts.

Another virulence property of fungal pathogens i.e. drug resistance, has come to the forefront only very recently [55–57].We recovered yeast isolates with high MICs for fluconazole and amphotericin B, and a narrow range of MIC against caspofungin and voriconazole. In addition, the MIC50 values of *C. krusei* and *C. tropicalis* for fluconazole were marginally higher than those reported in the clinical setting [58, 59]. Very high MIC 90 values for almost all *Candida* isolates as shown in this study are of major concern. It is a common practice in commercial poultry to add growth promoters and antimicrobials to poultry feed to protect the poultry from various diseases. We hypotheticise that these practices could be contributory towards such high level of drug resistance. At the same time, the intrinsic resistance of various yeasts to these antifungal agents cannot be overlooked. It is important to determine the exact sources of resistance development in poultry yeasts in order to develop strategies to arrest their propagation. It warrants careful monitoring as these drug-resistant strains could be of human origin [60] that might have evolved resistance due to antimicrobial pressure and subsequently disseminated to the domesticated flocks. It is difficult to conclusively prove this proposition, without demonstration of shared phenotypic and/or genotypic markers between human and poultry isolates. This necessitates further molecular studies.

It is well known that horizontal gene transfer among yeasts and zoonotic transmission of yeasts to humans is rare. Our study supports the hypothesis that poultry birds could be potential reservoirs of virulent and drug-resistant yeasts. Based on our and other reports [2, 3, 5, 8, 9, 42–45] we propose further studies to evaluate in-vivo virulence and clonal similarities between poultry and human isolates. Such studies will determine the potential of poultry yeasts to migrate to humans and vice versa*.*

Conventional culture based methods are beset with challenges in recovering the large spectrum of colonizing yeast in poultry gut flora. Advanced culturing methodologies and sequencing technology may be helpful in profiling both cultivable and non-cultivable mycobiome in poultry.

## Conclusion

The present work extends the current understanding of phenotypic characteristics of normal gastrointestinal yeast flora of *G domesticus* by providing information on in vitro virulence factors and antifungal susceptibility pattern. It is, therefore, reasonable that future studies should pay much attention in assessing the possible risk of transmission to humans or animals due to the dissemination of yeasts via food chain or environmental routes.

## Abbreviations

AMB: Amphotericin B deoxycholate
ATCC: American Type Culture Collection
BSA: Bovine Serum Albumin
CFG: Caspofungin
CFU: Colony forming units
CLSI: Clinical and Laboratory Standards Institute
CSH: Cell Surface Hydrophobicity
DNAse: Deoxyribonuclease
FLC: Fluconazole
GIT: Gastrointestinal tract
IGS1: Intergenic spacer 1
IQR: Interquartile range
MIC: Minimum Inhibitory Concentration
PBS: Phosphate Buffered Saline
rDNA: Ribosomal DNA
SAS: Statistical Analysis System
SDA: Sabouraud Dextrose Agar
SOD: Superoxide Dismutase
VRC: Voriconazole
Vs: Versus
XTT: sodium 39-[1-(phenylamino-carbonyl)-3,4-tetrazolium]-bis (4-methoxy-6-nitro) benzene sulfonic acid hydrate
YEPD: Yeast Extract Peptone Dextrose Agar
